# Role of nutrient supplements in children with post-COVID condition: a retrospective preliminary observation and narrative review

**DOI:** 10.1186/s13052-025-01961-5

**Published:** 2025-04-16

**Authors:** Rosa Morello, Cristina De Rose, Laura Martino, Francesca Raffaelli, Giuseppe Zampino, Piero Valentini, Danilo Buonsenso

**Affiliations:** 1 Department of Woman and Child Health and Public Health, Fondazione Policlinico Universitario A. Gemelli IRCCS, Rome, Italy; 2 Department of Laboratory and Infectivology Sciences, Fondazione Policlinico Universitario A. Gemelli IRCCS, Rome, 00168 Italy; 3 Centro di Salute Globale, Università Cattolica del Sacro Cuore, Roma, Italia; 4 Dipartimento di Scienze della Vita e di Sanità Pubblica, Area Pediatrica, Università Cattolica del Sacro Cuore, Roma, Italia

**Keywords:** SARS-Cov-2 infection, Long COVID, Post COVID condition (PCC), Children, PCC treatment, Nutrient supplements

## Abstract

**Background:**

Post-COVID Condition (PCC), emerging as a significant long-term consequence of SARS-CoV-2 infection, affects not only adults but also the pediatric population. Despite ongoing research, the precise pathophysiology of PCC remains elusive. However, several putative mechanisms have been identified, leading to the exploration of various therapeutic strategies. Notably, in the adult population, there has been substantial interest in the potential efficacy of nutritional supplements. Regrettably, information regarding the use of such supplements in the pediatric population is currently lacking.

**Methods:**

The present study was conducted to assess the impact of nutritional supplements on alleviating long COVID symptoms in children. To achieve this, we conducted a retrospective analysis of nutrient supplements administered by parents to children with Post-COVID Condition (PCC) between February 2020 and October 2022. Statistical analyses were employed to determine associations between categorical variables.

**Results:**

A total of 1243 children were enrolled following documented SARS-CoV-2 infection, with 940 (76.2%) diagnosed as recovered and 294 (23.8%) diagnosed with Long COVID. Among Long COVID patients experiencing disabling symptoms, treatment with oral lactoferrin and/or a Multi-Element Product (MEP) with antioxidant and anti-inflammatory properties was initiated. The correlation analysis between the use of supplements and persistence of long COVID at the next follow-up showed that the use of MEP alone (OR 5.7, 95% CI 3.8–8.5), or the combination of MEP and lactoferrin (OR 5.06, 95% CI 3.3–7.6) three months after the initial infection and for the following three months, were associated with a lower risk having long covid at six months following initial infection, when compared with the use of lactoferrin alone (OR 7.6 95% CI 5.1–11.4).

**Conclusions:**

This proof-of-concept study revealed that MEP and lactoferrin, when administered three months after initial infection in patients with a new diagnosis of long covid, may have a positive impact on improving Long COVID symptoms in children during follow-up evaluations. This positive trend toward reducing Post-COVID Condition (PCC) exhibited by MEP and lactoferrin suggested a potential benefit worthy of exploration in future randomized controlled trials.

**Supplementary Information:**

The online version contains supplementary material available at 10.1186/s13052-025-01961-5.

## Introduction

Post-COVID Condition (PCC) is a long-term consequence of SARS-Cov2 infection that can affect both adults and children [1]. Estimates suggest that about 40% of patients after an acute infection develop at least one of the symptoms of long COVID syndrome [2]. In the pediatric population, long COVID appears to afflict approximately one-quarter of children, with symptoms persisting even one year post-infection [3]. In most children, more than one organ system is involved, especially the respiratory, cardiovascular, neuromuscular systems, and brain [4]. Given the significant impact of PCC on global health, particularly concerning children’s well-being, there is a keen interest within the scientific community to identify its pathogenic mechanisms and develop corresponding therapeutic strategies. A heterogeneous entity with a broad spectrum of clinical manifestations, PCC paradoxically has a pathophysiology that is not yet precisely known. Putative mechanisms of pathogenesis are the persistence of SARS-CoV-2; reactivation of other viruses, in particular, herpesviruses as Epstein-Barr virus (EBV) and human herpesvirus 6 (HHV-6); autoimmunity; persistent tissue damage and immunity-triggered inflammation; microvascular thrombosis; impacts of SARS-CoV-2 on the microbiota; Impaired signaling within the brainstem and/or vagus nerve [5–7]. Aberrant myofibroblast proliferation and apoptosis/epithelial damage in the airway may be yet another factor responsible for fibrotic lung injury and respiratory disease [8]. The hypersensitization of peripheral and central neurons due to SARS-CoV2 neuroinflammation could be the cause of neurological symptoms, dysautonomia, and postural orthostatic tachycardia syndrome (POTS) [9, 10]. Viral persistence, mucosal and systemic immune dysregulation, microbial dysbiosis, insulin resistance, and metabolic abnormalities are linked to persistent gastrointestinal symptoms after SARS-Cov2 infection [11]. Augmented arginine and lipid metabolism (associated with the pro-inflammatory state) have been identified by metabolomic analyses in long COVID patients affected by chronic headache. Moreover, dysregulation of neurotransmitter systems (serotonin, dopamine, glutamate, and GABA) seems to occcur during long COVID headache [12]. Finally, mitochondrial dysfunction, changes in cellular energy metabolism, and reduced oxygen supply to tissues could be responsible for long COVID symptoms such as fatigue [10]. Given the mentioned candidate pathogenetic mechanisms, different strategies have been hypothesized as therapeutic possibilities: probiotics [13], vitamin supplements [14–16], lactoferrin [17], melatonin [18], flavonoids [19], anticoagulants [20], oxaloacetate [21], palmitoylethanolamide [22], omega-3 [23], coenzyme Q10 [24], aminoadamantanes [25], antiviral [26], and immunoglobulin [27]. Most studies are conducted on adults and available studies on the pediatric population are lacking in the literature. In light of these considerations, given the absence of side effects and the well-known anti-inflammatory and immunomodulatory effects of lactoferrin, vitamins, and minerals [28], we retrospectively analyzed the nutrient supplements used by parents of children with PCC and how they may have affected improvement or symptoms persistence.

## Materials and methods

### Study population and setting

This is a retrospective sub-analysis of a previously published prospective follow-up cohort of children after SARS-Cov-2 infection. The study population was a cohort of pediatric patients (< 18 years of age) who needed primary or secondary medical care for COVID-19 [29]. The study setting was a Post-COVID clinic in a third-level hospital in Rome, Italy. The authors developed a protocol to identify children with persistent symptoms after acute SARS-Cov-2 infection [28]. In case of the persistence of symptoms, patients underwent further examinations to exclude other diagnoses (such as anemia, celiac disease, autoimmunity, thyroid disease, etc.). In the absence of other possible causes, children with persistent symptoms for at least three months after the initial infection, which impaired daily life, were diagnosed as having post-COVID condition, according to the definition provided by Stephenson T et al. [30]. In particular, children were defined as having long covid if they had persisting symptoms lasting at least three months since initial infection and these symptoms had impact on daily activities (e.g. inability to attend school and/or practice the sports practiced before having Covid-19), and other potential diagnoses were excluded. The study was conducted from February 2020 to October 2022. All children were assessed at 3-6-12-18 months following SARS-Cov-2 acute infection, as part of a study that will include 24 and 36 months of follow-up. The same group of pediatricians in charge of the post-COVID clinic evaluated patients during follow-ups.

Initially in our Post-COVID Unit, in patients with persistent and disabling symptoms, oral lactoferrin and/or Multi-Element Product (MEP) were recommended (chewable tablets once a day for 90 days), based on the assessing physician decision. Lactoferrin’s complete composition is: Lactoferrin; corn maltodextrin; anticaking agents (magnesium salts of fatty acids, silicon dioxide.); capsule in hydroxypropyl methylcellulose.

MEP’s complete composition is: magnesium 200 mg, quercetin 150 mg, curcumin Meriva^®^100 mg, resveratrol 20 mg, vitamin E 15 mg, zinc 5 mg, folic acid 90 µg, selenium 55 µg, cholecalciferol 20 µg in one tablet.

### Inclusion and exclusion criteria

#### Inclusion criteria

- Children aged 0–18 years who were referred to our outpatient clinic during the study period.
- Cases confirmed with SARS-CoV-2 infection through laboratory testing (RT-PCR, COVID-19 antigen tests, or SARS-CoV-2 antibody testing).
- First assessment conducted 60 days after the diagnosis of COVID-19 infection.
- Consent obtained from the parent(s)/caregiver(s)/guardian(s) for participation in the study.
- Patients received either lactoferrin or MEP or both as supportive treatments for their long covid.

#### Exclusion criteria

- Patients ≥ 18 years.
- Children referred to our outpatient clinic outside the designated study period.
- Cases with suspected SARS-CoV-2 infection lacking laboratory confirmation.
- Absence of consent from the parent(s)/caregiver(s)/guardian(s) for participation in the study.
- Patients did not receive neither lactoferrin or MEP as supportive treatments for their long covid, or received them but also used other therapies.
- Patients were excluded if during the follow-up had other infections with SARS-CoV-2 or other viruses.

### Outcome

Outcome of this study was to evaluate the changes in long covid diagnoses at six months following initial infection in children diagnosed with long covid at three months after initial infection and treated with either lactoferrin, or MEP, or both. Patients were defined as cured from long covid when they had no more persisting symptoms and returned to daily pre-covid activities.

### Statistical analysis

For continuous variables, visual inspection of Q-Q plots and P-P plots were used to assess whether the distribution was normal or not. Categorical variables were reported as count and percentage. Continuous variables with normal distribution were expressed as mean with standard deviation; non-normal data were expressed as median with interquartile range (IQR 25-75%). The statistical association between categorical variables was obtained by Chi-squared tests or Fisher’s exact tests. Mann-Whitney U-test was used to assess differences in two groups for continuous variables if not normally distributed. P value < 0,05 was considered statistically significant. Statistical analysis was performed using IBM SPSS Statistics 28.0 software (IBM Corporation, Armonk, NY, USA).

### Ethical approval

The study is part of a larger prospective follow-up of children with Long COVID and was approved by the local institutional ethics review committee (Ethical Approval ID 4518, Protocol 0040139/21). Written and informed consent was obtained from parents/caregivers and children older than 5 years of age, in accordance with the local guidelines of the ethics committee.

## Results

During the study period were enrolled 1243 patients (575 (46,3%) were female). The full details of the study population have been reported elsewhere [29]. At 3 months 940 (76,2%) were diagnosed as recovered, and 294 (23,8%) children were diagnosed as Long COVID patients. Long COVID patients were 143 (12.2%), 38 (22.8%), and 15 (19.5%) respectively at 6, 12, and 18 months of follow-up. The main persisting symptoms at three months following initial SARS-CoV-2 infection in the study population and according to the treatments prescribed at three months follow-up are reported in Table 1.

**Table 1:** Prevalence of symptoms’ persistence at three months in the whole study population and according to treatments subsequently prescribed

	All 1250	MEP + LF 152	LF 18	MEP 9	*P* value
**Mean Age (y)**	6.8	7.1	6.9	7.1	> 0.05
**Persisting Symptoms**					
**Low grade fever**	8 (0.6)	2 (1.3)	2 (11.1)	0	> 0.05
**Anosmia**	11 (0.9)	5 (3.3)	1 (5.6)	1 (11.1)	> 0.05
**Dysgeusia**	11 (0.9)	4 (2.6)	2 (11.1)	0	> 0.05
**Chronic Cough**	16 (1.3)	5 (3.3)	1 (5.6)	0	> 0.05
**Dyspnea with mild efforts**	77 (6.2)	38 (25)	9 (50)	3 (33.3)	> 0.05
**Tachycardia**	20 (1.6)	12 (7.9)	1 (5.6)	1 (11.1)	> 0.05
**Joint Pains**	31 (2.5)	14 (9.2)	5 (27.8)	1 (11.1)	> 0.05
**Muscle Pains**	53 (4.2)	28 (18.4)	8 (44.4)	0	> 0.05
**Chronic Headache**	69 (5.5)	43 (28.3)	8 (44.4)	2 (22.2)	> 0.05
**Chronic gastrointestinal problems**	56 (4.5)	30 (19.7)	4 (22.2)	1 (11.1)	> 0.05
**Rashes**	14 (1.1)	7 (4.6)	1 (5.6)	1 (11.1)	> 0.05
**Easily fatigued with mild efforts**	15 (1.2)	6 (3.9)	1 (5.6)	0	> 0.05
**Concentrations and Memory difficulties**	31 (2.5)	19 (12.5)	2 (11.1)	0	> 0.05
**Unusual Fatigue**	162 (13)	93 (61.2)	11 (61.1)	3 (33.3)	> 0.05
**Chest pain**	29 (2.3)	14 (9.2)	2 (11.1)	1 (11.1)	> 0.05

MEP: Multi-Element Product

LF: lactoferrin

Nutraceutical products (lactoferrin and MEP), were prescribed to patients with persistent and disabling symptoms at least three months after acute infection. MEP was prescribed at 161 (13,0%), 53 (4,5%), 9 (5,4%), and 3 (3,9%) patients respectively to 3, 6, 12, and 18 months of follow-up. Lactoferrin was prescribed at 170 (13,8%), 48 (4,1%), 10 (6,0%), and 3 (3,9%) patients respectively to 3, 6, 12, and 18 months of follow-up. Both lactoferrin and MEP were prescribed at 152 (12,3%), 48 (4,1%), 9 (5,4%) and 3 (3,9%) patients respectively to 3, 6, 12, and 18 months of follow-up.

The correlation analysis between the use of supplements and persistence of long COVID at the next follow-up (Table 2) showed that the use of MEP alone (OR 5.7, 95% CI 3.8–8.5), or the combination of MEP and lactoferrin (OR 5.06, 95% CI 3.3–7.6) three months after the initial infection and for the following three months, were associated with a lower risk having long covid at six months following initial infection, when compared with the use of lactoferrin alone (OR 7.6 95% CI 5.1–11.4). No statistically significant associations were found between administration of MEP and/or Lactoferrin and Long Covid resolution when the therapy was started six months or longer after the initial infection.

**Table 2:** Odd ratios (OR) for persistence of long COVID at 6 months in children treated when diagnosed at three months following initial infection according to treatment with MEP or lactoferrin

	Six months follow-up (*n* = *1171*)	Twelve months follow-up (*n* = *167*)
MEP*, *OR (95% CI)*	5,7 (3,8–8,5)	2,71 (0,95 − 7,6)
Lactoferrin*, *OR (95% CI)*	7,6 (5,1–11,4)	2,07 (0,63 − 6,4)
MEP and Lactoferrin*, *OR (95% CI)*	5,06 (3,3–7,6)	2,03(0,63 − 6,4)

*prescribed at three months follow-up

## Discussion

The present study was performed to evaluate the role of MEP and lactoferrin in the reduction of symptoms in a cohort of long COVID pediatric patients assessed by multiple FUPs in a third-level hospital in Rome, Italy. To our knowledge, this is the first study that investigates the role of nutrient supplements in children with PCC, showing a potential utility, to be confirmed in trials, of the association of MEP and lactoferrin when prescribed at three months following the initial SARS-CoV-2 infection (being the first timepoint useful to diagnose long covid, as symptoms are requested to last at least 8–12 weeks for the diagnosis). However, no statistically significant associations were found between administration of MEP and/or Lactoferrin and Long Covid resolution when the therapy was started six months or longer after the initial infection, suggesting that a similar therapy may have beneficial effects only if started three months after initial infection.

During the recent COVID-19 pandemic, studies have revealed that deficiencies in Vitamin D, zinc, selenium, and magnesium are associated with heightened inflammatory responses and poorer prognoses in acute SARS-CoV-2 infection [31]. Moreover, emerging evidence suggests a link between nutritional deficits and long-term COVID-19 complications, indicating that nutritional deficiencies may underlie the syndrome’s development [32]. A UK study observed a high prevalence of vitamin D deficiency among children with Pediatric Inflammatory Multisystem Syndrome Temporally Associated with SARS-CoV-2 (PIMS-TS) [33], while Schloss JV [34] proposed that nutritional deficits could predispose individuals to long COVID. Consequently, with the putative pathogenesis of Post-COVID Condition (PCC) in mind, researchers have speculated about the potential protective role of nutrient supplements. These supplements may not only help prevent infection and support immune responses during acute COVID-19 but also mitigate inflammation and oxidative stress during the long COVID phase [14–16].

Vitamin D has a known immune-modulating effect and can prevent the frequency of infections, especially respiratory infections compared to placebo [35, 36]. Pak VM et al. [37] in their systematic review suggested that vitamin D could reduce inflammation and improve fatigue symptoms in long COVID [37, 38]. However, whether patients with PCC exhibit low levels of vitamin D [39] is still debated [39]. Magnesium (Mg) plays essential roles in the regulation of cell growth, division, differentiation, redox homeostasis, and reduction of inflammation [40] and might contribute to protecting and reducing the severity of the acute SARS-CoV-2 infection and facilitate recovery after the acute phase [41]. Coman AE et al. [42] underlined that magnesium is involved in cognitive functions and is essential for all muscle enzymes. Furthermore, the authors reminded that magnesium deficiency usually causes psychiatric symptoms (anxiety, insomnia, hyperemotionality, depression, headache, dizziness, and tremors), suggesting that magnesium could be involved in the development of long COVID-19 syndrome. The association of low serum Mg with a higher incidence of long COVID symptomatology was documented among 95 patients by La Carruba A. et al. [43].

Zinc is another essential micronutrient crucial for maintaining cellular physiologic homeostasis that plays indispensable roles in cellular processes ranging from growth and development to redox homeostasis and reduction of inflammatory cytokines and it may prevent latent virus reactivation [44]. Studies suggest that nutrient supplements containing zinc may decrease the expression of Interleukin-6 (IL-6) and alleviate myalgia in COVID-19 patients [45], as well as also potentially reducing the duration of post-infection anosmia [46] and improving sleep and fatigue in those experiencing the Post-COVID Condition (PCC). A recent retrospective observational study in Japan found that fatigue, dysosmia, and dysgeusia were the most common symptoms among long COVID patients with hypozincemia [47]. Selenium, another crucial micronutrient, plays a vital role in antioxidant defense systems and has been implicated in not only protecting endothelial functions [32]. but also has been suggested to play a significant anti-inflammatory role in long COVID [48, 49].

The B-group vitamins represent essential micronutrients for energy metabolism, DNA and protein synthesis, and immune cell regulation [32]. A study by Sousa et al. [50] documented improvement in post-COVID olfactory dysfunction after Vitamin B complex administration along with other treatments (olfactory training/topical corticosteroid). Natural substances such as resveratrol, quercetin, sulforaphane, and curcumin could limit the effect of the cytokine storm and persistent inflammation and autoimmunity. Moreover, polyphenols and flavonoids have antiarrhythmic and antiplatelet properties. Curcumin reduces oxidative stress, inflammation, and pain, and has neuroprotective effects [32]. Flavonoids have antioxidant, anti-inflammatory, antiviral, antibacterial, and anti-cancer properties. Melsore J et al. suggests that dietary supplements or diets rich in flavonoids can be beneficial in the treatment of PCC [51]. Ter Ellen BM et al. provide evidence that resveratrol had potent antiviral properties against SARS-CoV-2 in vitro [52]. Tosato M et al. [53] documented the role of l-arginine plus vitamin C supplementation in improving symptoms in long COVID patients.

In the existing literature, numerous studies have explored the efficacy of nutrient supplements in addressing post-COVID conditions, primarily focusing on adult patients. Naureen Z et al. in a pilot observational study, have advocated for the use of food supplements, such as hydroxytyrosol, acetyl L-carnitine, and vitamins B, C, and D, in the management of post-COVID conditions [54]. Their research indicates the potential for these supplements to alleviate perceived fatigue among patients experiencing post-COVID syndrome.

Lastly, several clinical trials have been conducted to investigate the effectiveness of nutrient supplements as potential treatments for long COVID. Since the iron-binding proteins lactoferrin has several known antimicrobial, anti-inflammatory, immunomodulating, and antioxidant properties, we proposed its use with a Multi-Element Product (MEP) as a preventive or therapeutic strategy for COVID-19 disease and long COVID, in particular for mitigation of the gastrointestinal manifestations of these disorders in children [17]. Our results, indeed, found possible benefits from MEP alone, or in association with lactoferrin, when administered at three months following the initial infection. Possible reasons for a greater effect of MEP compared with lactoferrin may be due to the more complex composition of the product, which contains several elements with well established biological effects, therefore potentially tackling multiple biological pathways that can be implicated in long covid, as previously described. Importantly, children that used the different medications were similar from a demographic and clinical characteristics, therefore it is plausible that the effect may be due to medication rather than different patient characteristics. Nevertheless, our study does not support the routine use of these nutrient supplements out of trials, but highlights potential benefits which should be explored in specifically designed randomized controlled trials.

### Strengths and limitations

The main limitation of this study is its retrospective nature. which limits the ability to establish causality, and the potential impact of selection bias, recall bias and other biases that may have affected results. In addition, all patients with persistent, disabling symptoms were treated, without a control group. Therefore, we cannot define if the improving trend observed with MEP is due to medication or spontaneous improvement of symptoms over time. Another consideration might be that lactoferrin was used as a therapeutic strategy already in children infected with the pre-omicron variant, which was an independent risk factor for persistent symptoms [29]. In addition, multivitamins were given as supportive treatment, but the patients’ initial vitamin levels are unknown. The effect obtained may be due to baseline vitamin insufficiency, either pre-existing or triggered by the initial viral infection. Last, not a sufficiently high number of patients were treated with one of the different pharmacological approaches at the other timepoints, therefore we could limit our analyses only to patients diagnosed and treated at 3 months following initial infection.

## Conclusions

In conclusion, in our proof-of-concept study, we found that MEP and lactoferrin, when administered three months after initial infection in patients with a new diagnosis of long covid, may have a positive impact on improving Long COVID symptoms in children during follow-up evaluations. This positive trend toward reducing Post-COVID Condition (PCC) exhibited by MEP and lactoferrin suggested a potential benefit worthy of exploration in future randomized controlled trials, given the biologically plausible effects of these compounds (Table 3). Further studies, in larger populations and with control groups, would be needed to determine the therapeutic role of these products in children, either in the treatment of PCC or better as an early treatment [56] in children at risk of developing PCC as a preventive therapeutic approach.

**Table 3:** Biological effects of micronutrients and lactoferrin. Adapted from Buonsenso et al. [23] and Mangoni et al. [55]

	Antiviral activity	Immune modulation	Anti inflammatory	Autoimmunity prevention	Anti oxidand	Anti thromobotic	Endothelial protective	Cyto protective & organ damage prevention	Anti arrhythmic	Anti depression	Microbiome
Folic Acide	-	Yes	Yes	-	Yes	Yes	Yes	Yes	-	Yes	Yes
Vitamin C	-	Yes	Yes	?	Yes	-	-	-	-	-	-
Vitamin D	Yes	Yes	Yes	Yes	Yes	Yes	Yes	Yes	Yes	Yes	Yes
Vitamin E	-	Yes	Yes	?	Yes	Yes	Yes	Yes	-	-	Yes
Magnesium	-	Yes	Yes	?	Yes	Yes	Yes	Yes	Yes	?	-
Seleium	Yes	Yes	Yes	Yes	Yes	Yes	Yes	Yes	?	?	?
Zinc	Yes	Yes	Yes	Yes	Yes	-	Yes	-	-	Yes	?
Phyto chemicals	Yes	Yes	Yes	Yes	Yes	Yes	Yes	Yes	Yes	Yes	Yes
Lactoferrin	Yes	Yes	Yes	-	Yes	-	-	-	-	-	Yes

## Electronic supplementary material

Below is the link to the electronic supplementary material.

Supplementary Material 1

## Data Availability

Available upon request to the corresponding author.

## Abbreviations

PCC: Post covid condition
MEP: Multi-Element Product
