# Supplementary material for: Role of nutrient supplements in children with post-COVID condition: a retrospective preliminary observation and narrative review
Source: Ital J Pediatr. 2025 Apr 16;51:119. doi: 10.1186/s13052-025-01961-5 (PMC12004621; doi:10.1186/s13052-025-01961-5)
Supplement: Supplementary file 1 — Supplementary Material 1 [file 13052_2025_1961_MOESM1_ESM.docx]

Dear Editor and Reviewers

Thank you very much for your suggestions, which helped us to significantly improve our paper.

Please find below a point-by-point response to your comments. Changes have been highlighted within the manuscript

Reviewer reports:

Reviewer #1: Thank you for the opportunity to review this interesting research that sheds light on the use of nutritional supplements for the treatment of long-COVID, a currently relevant issue, in a population where data is often lacking. I raise the following points for the authors' consideration.

*Thank you for finding our paper interesting and for your detailed revisions, they helped in significantly improve our paper*

Introduction and general points
1.      The overall structure could be improved. For instance, the outcome section of the methods reads more like an objective than an endpoint/outcome of the study. The objective could be stated only one time at the end of the introduction or at the beginning of the method section instead, and the outcomes subheading could be reformulated to include the specific endpoints that were used: what were the exact symptoms or criteria used to measure "improvement" or "resolution"? Was improvement measured using standardised tools, parent-reported outcomes, or subjective clinical assessments?

*Thank you very much, we entirely agree with this point and changed accordingly, as follow: Outcome of this study was to evaluate the changes in long covid diagnoses at six months following initial infection in children diagnosed with long covid at three months after initial infection and treated with either lactoferrin, or MEP, or both. Patients were defined as cured from long covid when they had no more persisting symptoms and returned to daily pre-covid activities.*

2.      Some or all of the tables could be included in the results section since they are not so large to impair the reading flow.

*We put them in a separate file as per editorial rules, sorry*

3.      A separate paragraph titled "strengths and limitations" enriched with details suggested below would make the manuscript more structured and complete.

*Thank you, we have added it*

4.      The English language used is at times redundant and at times could be improved (see for example lines 2, 29 page 2; 8-9 and 13-14 page 3; 10-11 and 43-44 page 4; 14 page 5; 55-56 page 6). I would also suggest referring to the treatments discussed using the same expression consistently throughout the manuscript, and check that the abbreviation used is explained only one time, after its first use. If you choose to use the abbreviated form 'NS', use it every time except for the first time when the full expression is to be reported. Alternatively, you could avoid the short form altogether and consistently use the full expression "nutraceutical products", "nutrient supplements", or "nutritional supplements".

*We agree and we used the full expression consistenly*

5.      Consider justifying the focus on lactoferrin and the Multi-Element Product (MEP) as the primary supplements under investigation, considering that many other supplements have similar anti-inflammatory properties and low adverse effect profiles. Additionally, given the retrospective nature of the study, it would need to be clarified whether children taking other supplements were excluded from the sample and, if so, how these exclusions were handled.

*Thank you, we have clarified that only those receiving either lactoferrin or MEP or both were included in the study, and those not receiving them or also using other therapies were excluded. The rational for using those medications as supportive therapies was justified in the introduction.*

Methods
1.      The possibility of interaction effects between lactoferrin and MEP if and when they were used together does not seem to have been addressed, raising questions about the possibility to isolate the effects of each supplement independently. Did some children receive both supplements? And, if so, would it be feasible to conduct separate analyses for children receiving only one or the other and children receiving both supplements? If stratified analysis or interaction testing was not feasible, could this limitation be discussed explicitly in the discussion?

*Thank you for this comment. Indeed, some used both, and therefore, following your suggestion, we re-performed all the analyses, including a third group of patients receving both of them and comparing them from a statistical perspective.*

2.      Were the supplements prescribed by doctors or administered by parents of their own initiative? In the methods section, the expression "administered by parents" is used, while later on, it is mentioned that oral lactoferrin and/or MEP were prescribed. If supplements were prescribed by healthcare providers, what criteria were used to decide which children would receive lactoferrin, MEP, or both? Were supplements offered to all children with long COVID or only some? Did all families accept this therapeutic option or did some refuse? Was the parallel use of other medication, whether related to long COVID or not, considered/excluded? Was the possible occurrence of other infections during follow-up considered/excluded? Could children who received supplements differ from those who did not in ways that could affect outcomes (i.e., severity of symptoms, socioeconomic status, or parental concern)?

*We clarified that “Initially in our Post-COVID Unit, in patients with persistent and disabling symptoms, oral lactoferrin and/or Multi-Element Product (MEP) were recommended (chewable tablets once a day for 90 days), based on the assessing physician decision.” In the methods, we clarified that those taking other therapies or having reinfections or other viral infections were considered exclusion criteria.*

3.      The stated inclusion criteria are broadly defined as children aged 0-18 who were referred to the clinic and had confirmed SARS-CoV-2 infection. However, the study focuses on a subgroup of children with long COVID, specifically those who received supplements. It would be helpful to further detail the listed inclusion and exclusion criteria accordingly (long COVID diagnosis and supplement use) and to add a flow diagram of the population selection process, including numbers of participants lost at each stage of selection, if applicable.

*Thank you, as stated before we added this inclusion criteria for further clarification*

4.      Despite the reference to both a protocol to identify children with persistent symptoms and a diagnosis of post-COVID condition "according to the definition provided by Stephenson et al.", it might be helpful to explicitly outline in the main text of this study the fundamental aspects of both the resources mentioned, clarifying how these were used to select children for inclusion in the study. For instance, the specific symptoms that needed to be persistent for at least three months in order to confirm a long COVID diagnosis are not outlined. Even if these are included in the referenced larger study, a brief overview would improve clarity and transparency.

*Thank you, we clarified it: In particular, children were defined as having long covid if they had persisting symptoms lasting at least three months since initial infection and these symptoms had impact on daily activities (eg inability to attend school and/or practice the sports practiced before having Covid-19), and other potential diagnoses were excluded.*

Discussion
1.      Possible reasons as to why MEP shows a trend toward benefit at later rather than earlier assessments might be worth commenting on, as well as the possible reasons why lactoferrin may have adverse associations (could it have been used in children with more severe manifestations, making the use of lactoferrin a confounding variable?)

*Thank you very much, we clarified this point as follow: “Our results, indeed, found possible benefits from MEP alone, or in association with lactoferrin, when administered at three months following the initial infection. Possible reasons for a greater effect of MEP compared with lactoferrin may be due to the more complex composition of the product, which contains several elements with well established biological effects, therefore potentially tackling multiple biological pathways that can be implicated in long covid, as previously described. Importantly, children that used the different medications were similar from a demographic and clinical characteristics, therefore it is plausible that the effect may be due to medication rather than different patient characteristics.”*

2.      The limitation section could be expanded to include comments on the retrospective nature of the study, which limits the ability to establish causality, and the potential impact of selection bias, recall bias and other biases that may have affected results. The lack of statistical significance warrants a clearer statement on how results are to be interpreted with caution. While the need for an RCT is acknowledged, a more detailed examination of the study's specific limitations and gaps could help propose targeted hypotheses or study designs to address these challenges.

*Thank you, we have added the mentioned limitations, as we agree with you. Importantly, we have redone the statistical analyses as suggested by reviewers, which significantly improve our study.*

3.      The concluding statement in the discussion on the use of supplements as an early treatment or preventive approach in children at risk of PCC, however referenced, is not supported by the results of this research. I suggest either commenting further on how the results discussed might support this final point or maintaining the focus of the conclusion on the study results.

*We entirely agree with you, we have removed that statement.*

Reviewer #2:
I read the article titled "Role of nutrient supplements in children with Post-COVID condition: a retrospective preliminary observation and narrative review" with interest. I list my suggestions below:

*Thank you for your interest and your suggestions*

1- The article was designed retrospectively, but patients were given oral supplements such as lactoferrin prospectively. The method section should be written in detail and errors should be corrected.

*Thank you, methods have been expanded based on both reviewers comments*

2- Multivitamins were given as supportive treatment, but the patients' initial vitamin levels are unknown. The effect obtained may be due to insufficiency. This should be detailed in the discussion.

This is a good point, we have added it in the limitation. Thank you

3- Which patients were given lactoferrin, which patients were given mep? How was the randomization done?

*This was a retrospective study, as we clearly acknowledged, also in the discussion. Therefore, there was no randomization. However, as you suggested, we added information on the groups of patients based on treatments received, and they were statistically similar each other.*

Thank you for your help

Danilo Buonsens

On behalf of co-authors
